# Association between body mass index and prevalence of bacterial vaginosis: Results from the NHANES 2001–2004 study

**DOI:** 10.1371/journal.pone.0296455

**Published:** 2024-05-31

**Authors:** Jie Qi, Hua Han, Xinjun Li, Yanan Ren

**Affiliations:** Department of Gynecology, Hebei General Hospital, Shijiazhuang, China; UFRN: Universidade Federal do Rio Grande do Norte, BRAZIL

## Abstract

**Background:**

The impact of bacterial vaginosis on women’s health is an increasing concern; however, the effect of the obesity index on bacterial vaginosis is controversial. We investigated the association between body mass index and bacterial vaginosis in women in the United States.

**Methods:**

This was a cross-sectional study which obtained the data from the National Health and Nutrition Examination Survey from 2001 to 2004, in which weighted multivariate regression and logistic regression analyses were performed to explore the independent relationship between body mass index and bacterial vaginosis. Subgroup analyses and smoothed curve fitting were also performed.

**Results:**

A total of 5,428 participants were enrolled, and the findings show that the participants with higher body mass index tended to have a higher incidence of bacterial vaginosis. In the fully adjusted model, a positive association between bacterial vaginosis and body mass index was observed (Odd’s ratio = 1.03, 95% Confidence interval, 1.01–1.04). The subgroup analysis showed that this positive association was significant in non-Hispanic White individuals (Odd’s ratio = 1.0327, 95% Confidence interval, 1.0163, 1.0493).

**Conclusion:**

Increased bacterial vaginosis positivity may be associated with an increased body mass index.

## Material and methods

### Data source

The data used in this analysis were obtained from the National Health and Nutrition Examination Survey (NHANES), an ongoing survey aimed at assessing the health and nutritional status of individuals in the United States. Specifically, we used nationally representative data from the 2001–2004 cycles of NHANES. It is important to note that data on bacterial vaginosis (BV) were available only for the 2001–2002 and 2003–2004 cycles. This study used a cross-sectional design.

### Study population

Our study utilized population-based data from the NHANES. This survey included interviews and medical examinations focusing on various health and nutrition measurements. All participants provided written informed consent and the study was approved by the NCHS Research Ethics Review Board. The inclusion criteria were as follows: Women who participated in the NHANES 2001–2004 and had available Nugent score and body mass index (BMI) records (BMI <40 kg/m^2^). Exclusion criteria: Participants without BMI or Nugent score records. For more information, please refer to the official NHANES website (https://www.cdc.gov/nchs/nhanes/index.htm).

### Variables

#### Bacterial vaginosis

The BV scores were calculated using Nugent’s method. The final BV scores were categorized as follows: scores of 0–3 indicating normal vaginal flora, 4–6 indicating intermediate flora, and 7–10 indicating bacterial vaginosis [1]. The outcomes were defined as BV-confirmed yes (positive and intermediate) or no (negative).

#### Body mass index

BMI is a measure of body weight relative to height and is calculated as weight (in kilograms) divided by the square of height (in meters). In our analysis, the BMI was treated as a continuous variable.

#### Social demographic data

Social demographic data included age (in years), race (categorized as 1: Non-Hispanic White, 2: Non-Hispanic Black, 3: Mexican American, and 4: other races), educational level (categorized as below high school, high school, and college degree or above), marital status (categorized as living with a partner [married] or living alone [widowed, divorced, or separated]), and household income to poverty ratio (PIR). Other health-related variables included smoking status (whether the participant had smoked at least 100 cigarettes in their lifetime) 、vaginal douching in the past six months (yes or no)、diabetes (The diagnostic criteria for diabetes are:1.doctor told you have diabetes, 2.glycohemoglobin HbA1c(%) > = 6.5, 3.fasting glucose (mmol/l) > = 7.0, 4.random blood glucose (mmol/l) > = 11.1, 5.Use of diabetes medication or insulin; Prediabetes: Hba1c: > = 5.7 and <6.5,FPG: 5.6–7.0), pregnant.

### Statistical analysis

To ensure the accuracy of our analysis, the sampling weight of the NHANES data was incorporated into all statistical analyses. Specifically, multivariate logistic regression was used to assess the association between BV and BMI, while adjusting for covariates. Continuous variables are presented as mean ± standard deviation, and categorical variables are presented as percentages. A multivariate logistic regression model with NHANES sampling weights was used for the three models. No covariates were adjusted for in Model 1. In Model 2, adjustments were made for age and race. In Model 3, adjustments were made for age, race, education level, household income ratio, marital status, smoking status, and vaginal douching in the past 6 months. In the sensitivity analyses, we converted from a continuous variable to a categorical variable to assess its robustness. Additionally, we utilized a generalized additive model (GAM) and smoothed curve fitting to account for potential nonlinear relationships between BMI and BV. The statistical software packages R (http://www.R-project.org) and Empower Stats (http://www.empowerstats.com) were used for data analysis. Statistical significance was set at P < 0.05.

## Introduction

The stability of vaginal microecology plays an important role in the health of the female reproductive tract [2]. In 2011, Ravel et al. performed a bacterial sequencing analysis of vaginal samples from 396 reproductive-aged women and revealed the existence of five dominant bacterial vaginal microbiota types [3]. Subsequently, they classified them into 13 types. Although racial differences exist in the vaginal microbiota, lactobacilli dominate the vaginal microbiota of healthy reproductive-aged women. A decrease in lactobacilli and an increase in vaginal microbial diversity have been linked to the acquisition and persistence of HPV as well as the development of cervical cancer [4, 5]. Furthermore, changes in the vaginal microecology can affect the endometrium [2].

Bacterial vaginosis is one of the most common types of vaginitis in women of reproductive age [6]. In BV, the dominant *Lactobacilli* is replaced by facultative anaerobes or anaerobic bacteria such as *Gardnerella vaginalis* and *Atobola vaginalis* in the vagina, resulting in an imbalance of normal flora and mixed infections (Klatt and Barnabei, 2010; Bradshaw and Sobel, 2016; Ranjit *et al*., 2018) [4, 7, 8]. If left untreated, BV can lead to complications, such as pelvic inflammatory disease, an increased risk of sexually transmitted infections, and preterm birth in pregnant women [9, 10].

Obesity is a global concern that poses significant health risks to individuals, and its impact on women should not be underestimated. Obesity can contribute to various gynecological complications, including irregular menstruation, polycystic ovary syndrome [11–13], infertility [14], hypertension during pregnancy [15], and gestational diabetes [16]. However, the effect of obesity on these outcomes is not fully understood. Although extensive research has been conducted on the effects of obesity on intestinal microecology, few studies have demonstrated its impact on vaginal microecology. Moreover, the association between obesity and BV remains controversial.

This study used the National Health and Nutrition Examination Survey (NHANES) database to evaluate the relationship between BV and BMI in American women.

## Discussion

In this cross-sectional study involving 5,428 participants, a positive correlation was found between BV and BMI, particularly among individuals of the Non-Hispanic White race. This suggests that an increase in BMI may contribute to a higher incidence of BV.

Previous studies on the relationship between BV and obesity have reported conflicting results. For instance, a prospective cohort study focusing on female sex workers in Mombasa, Kenya, found that obese women had a nearly 20% lower risk of BV than normal weight [17]. However, it is important to note that the Mombasa study differed significantly in population characteristics from the current NHANES cross-sectional study, which included women of various ethnicities. These racial differences were significant mainly because of the higher prevalence of BV in black women than in white women, thus indicating the presence of a more diverse vaginal microbiota in different ethnicities.

In a retrospective analysis, the researchers investigated the relationship between obesity and BV in 106 women of reproductive age. They concluded the there was no significant relationship between BMI and BV [18]. It is worth noting that this retrospective study only included 106 participants and did not take race into account, which may be the main reason for the difference with the results of the NHANES cross-sectional study.

Another retrospective analysis conducted by Daubert et al. included 10,184 women infected with HIV or at risk of infection. The study showed that the rate of BV in women with obesity (BMI >30 kg/m^2^) was lower compared to the reference group (BMI 18.5–24.9 kg/m^2^) (adjusted OR, 0.87: 95% CI, 0.79–0.97: P = 0.009). It is worth considering that this study focused on HIV-positive women, the differences in sexual behavior between HIV-positive patients and non-infected women may have influenced the results [19]. The current NHANES cross-sectional study did not consider HIV infection as a factor. Si et al. investigated the genetic relationship between vaginal microbiota and obesity in 542 Korean women and demonstrated that obesity was associated with an increase in *Prevotella* levels and a decrease in the relative abundance of lactobacilli [20]. This finding was inconsistent with our conclusions. Similarly, Raglan et al. conducted a prospective study on 109 women and discovered that obese women had a lower prevalence of lactobacilli-dominated vaginal microbiota and a higher prevalence of high-diversity vaginal microbiota (depleted in *Lactobacillus* spp. and *Gardnerella* spp.) when compared to non-obese participants (P < 0.001) [21]. Brookheart et al. conducted a cross-sectional study on 5,918 participants and observed a higher incidence of BV in overweight and obese women compared to thin women [22].

Obesity is characterized by a low inflammatory state, and the mechanisms that lead to an increased incidence of BV are likely multifaceted [23, 24]. First, obesity can contribute to gynecological diseases, such as menstrual irregularities and polycystic ovary syndrome [25]. Menstrual blood has a significant effect on vaginal microecology [25–27]. Second, obesity can affect personal hygiene habits and excess adipose tissue increases vaginal moisture, which may change the vaginal environment. Obesity can also affect glycogen levels in the vaginal epithelium, as it decreases estrogen levels rather than increasing them, resulting in a decrease in the abundance of *Lactobacillus* bacteria and an increase in the concentration of certain anaerobic bacteria [28]. Additionally, changes in intestinal microecology can influence vaginal microecology and dietary habits, as well as the intestinal microecology of obese individuals, which may contribute to synergistic changes in vaginal microecology [29].

This study has several strengths. First, the study was based on a large population from the NHANES database, which provided a more representative sample. This study also considered confounding factors to enhance the credibility of the results. However, this study has some limitations. As this was a cross-sectional study, a clear causality cannot be established. Furthermore, the collected data may not have been recent. Additionally, important comorbidities such as polycystic ovary syndrome was not included in the data collection, which limited the ability to control for confounding factors.

In conclusion, this study suggests that increased BMI is associated with a higher incidence of bacterial vaginosis. This positive correlation was particularly noticeable among Non-Hispanic White individuals. However, further large-scale prospective studies are needed to validate these findings.

## Results

In total, there were 5,428 women enrolled in the current study, including 3,106 BV-positive women (age: 32.44±9.35, BMI: 26.16±5.28) and 2,322 BV-negative women (age: 32.34±9.57, BMI: 25.20±5.28). There were significant differences in BMI, household income, race, education, smoking, vaginal douching within 6 months, diabetes and pregnant. BV-positive individuals had a greater BMI when compared to that of BV-negative individuals (Table 1). The association between BMI and BV is shown in Table 2. Our results showed that a higher BMI was associated with BV positivity. In the fully adjusted model, a positive association was observed between BMI and BV positivity (OR = 1.03, 95% CI: 1.01–1.04), which suggests that for every unit increase in BMI, the incidence of BV increased by 2.03%. BMI was further converted from a continuous variable to a categorical variable for the sensitivity analysis. The increase in BV positivity was not very significant compared to that in the group with a BMI of less than 18.5 (Table 2). As shown in Table 3, the positive correlation between BV and BMI may have been influenced by age and educational attainment.

**Table 1:** Baseline characteristics of participant.

Characteristic	BV		
	negative	positive	P value
Age, mean(sd),years	32.3050 ± 9.5692	32.4444 ± 9.3505	0.588099
BMI,mean(sd),kg/m2	25.1969 ± 5.0009	26.1618 ± 5.2770	<0.000001
Poverty-to-income ratio, mean(sd)	3.0935 ± 1.6818	2.8234 ± 1.6604	<0.000001
Race/Ethnicity (%)			<0.000001
Non-Hispanic White	80.8439	68.8613	
Non-Hispanic Black	16.0260	20.7384	
Mexican American	65.1974	61.8961	
Other race/ethnicity	8.6818	6.9757	
Education level (%)			0.000029
Less than high school	10.0948	10.3898	
High school	16.0260	20.7384	
More than high school	65.1974	61.8961	
others	8.6818	6.9757	
Marital status (%)			0.072891
Married and living with partner	57.4710	55.0497	
Living alone	42.5290	44.9503	
Smoking (%)			0.000311
Yes	35.6371	40.5489	
No	55.6812	52.4755	
NA	8.6818	6.9757	
Whether there has been vaginal douching in the past 6 months			<0.000001
Yes	12.8783	20.5623	
No	82.9727	71.6656	
NA	4.1489	7.7721	
Diabetes			0.002
No	87.08	96.281	
Pre-Diabetes	0.89	0.965	
Yes	2.029	2.507	
Pregnant			<0.001
No	87.037	90.019	
Yes	12.963	9.981	

Data in the table

For continuous variables: P-value was by survey-weighted linear regression。 For categorical variables: P-value was by survey-weighted Chi-square test。BMI, body mass index; NHANES, National Health, and Nutrition Examination Survey; SD, standard deviation. BV, bacterial vaginosis

**Table 2:** The association between BMI and BV.

Exposure	Non-adjusted model OR,95%CI	Minimally-adjusted model OR,95%CI	Fully-adjusted model OR,95%CI
BMI	1.03 (1.02, 1.04)	1.02 (1.01, 1.03)	1.03 (1.01, 1.04)
BMI			
<18.5	Ref	Ref	Ref
> = 18.5, <25	0.87 (0.65, 1.15)	0.86 (0.64, 1.14)	0.83 (0.59, 1.16)
> = 25, <30	1.12 (0.84, 1.50)	1.05 (0.78, 1.42)	1.03 (0.72, 1.47)
> = 30	1.28 (0.95, 1.72)	1.13 (0.83, 1.53)	1.15 (0.80, 1.64)
P for trend	<0.0001	0.0004	0.003

Non-adjusted model: no covariates were adjusted for.

Minimally-adjusted model: we only adjusted for age and race.

Fully-adjusted model: we adjusted for all covariates presented in Table 1. Abbreviation: BMI, body mass index; BV, bacterial vaginosis

**Table 3:** Subgroup analysis of the association between BMI and BV.

Subgroup	OR (95%CI)	P for interaction
Age		<0.0001
<20	1.1018 (1.0724, 1.1320)	
20–40	1.0117 (0.9955, 1.0282)	
≥40	0.9940 (0.9681, 1.0206)	
Race/ethnicity		0.2497
Non-Hispanic White	1.0327 (1.0163, 1.0493)	
Non-Hispanic Black	1.0095 (0.9823, 1.0374)	
Mexican American	1.0224 (0.9931, 1.0525)	
Other race/multiracial	1.0612 (1.0142, 1.1104)	
Education level		<0.0001
Less than high school	0.9880 (0.9481, 1.0297)	
High school	0.9777 (0.9483, 1.0081)	
More than high school	1.0188 (1.0018, 1.0362)	
others	1.1020 (1.0726, 1.1322)	

Age, race, BMI, Marital status, Poverty-to-income ratio, Whether there has been vaginal douching

in the past 6 months, smoking were adjusted. In the subgroup analyses, the model is not adjusted for the stratification variable itself.

Abbreviation: BMI, body mass index; BV, bacterial vaginosis

**Fig 1:**
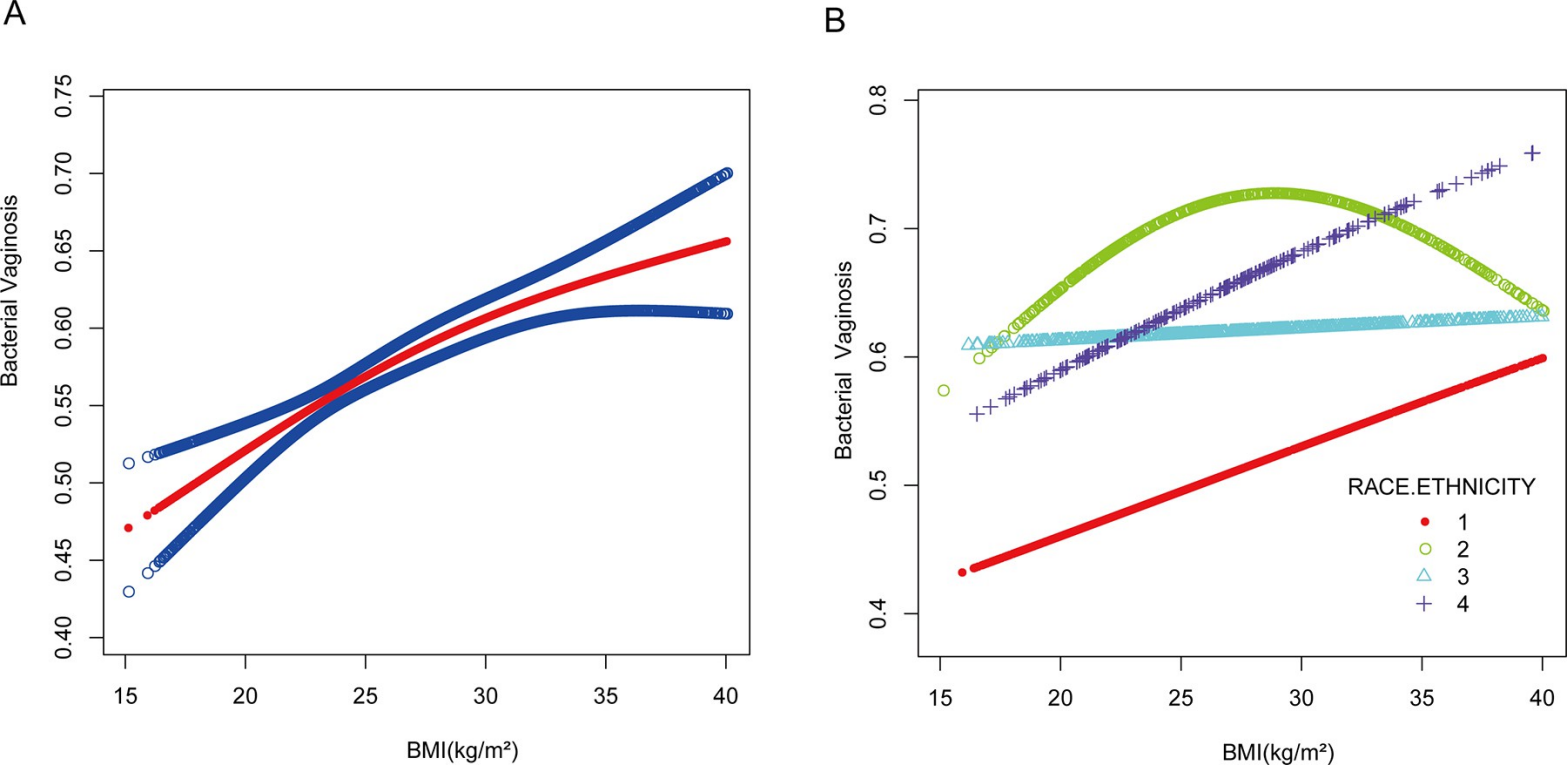
The association between BMI and BV. **A)** Solid line plot of curve fitting with BV and bmi as variables. The red line indicates the smooth curve fit between the variables. The 95% confidence interval of the fit is shown by the blue bar. (B) Curve-fit solid line plot of the relationship between BV and BMI analysed stratified by race.1: Non-Hispanic White,2: Non-Hispanic Black,3: Mexican American,4: Other race/ethnicity.

The current results suggest that the association between BV and BMI is not consistent across all races, and the authors observed a positive association between BV and BMI in whites stratified by race. Its curvilinear relationship was also positive, whereas this positive association was not particularly evident among Blacks and Mexicans (Table 3, Fig 1B). Smoothed splines were used to demonstrate the relationship between BMI and BV (Fig 1A).

## References

[1] Nugent et al. - 1991—Reliability of diagnosing bacterial vaginosis is i.pdf. 10.1128/jcm.29.2.297-301.1991 PMC269757 1706728

[2] Wang J, Li Z, Ma X, Du L, Jia Z, Cui X, et al. Translocation of vaginal microbiota is involved in impairment and protection of uterine health. Nat Commun. 2021;12: 4191. doi: 10.1038/s41467-021-24516-8 34234149 PMC8263591

[3] Ravel J, Gajer P, Abdo Z, Schneider GM, Koenig SS, McCulle SL, et al. Vaginal. microbiome of reproductive-age women. Proceedings of the National Academy of Sciences of the United States of America. 2011 Mar 15;108 Suppl 1(Suppl 1):4680–7. doi: 10.1073/pnas.1002611107 20534435 PMC3063603

[4] Bradshaw CS, Sobel JD. Current Treatment of Bacterial Vaginosis—Limitations and Need for Innovation. J Infect Dis. 2016;214: S14–S20. doi: 10.1093/infdis/jiw159 27449869 PMC4957510

[5] Mitra A, MacIntyre DA, Marchesi JR, Lee YS, Bennett PR, Kyrgiou M. The vaginal microbiota, human papillomavirus infection and cervical intraepithelial neoplasia: what do we know and where are we going next? Microbiome. 2016;4: 58. doi: 10.1186/s40168-016-0203-0 27802830 PMC5088670

[6] Kenyon C, Colebunders R, Crucitti T. The global epidemiology of bacterial vaginosis: a systematic review. American Journal of Obstetrics and Gynecology. 2013;209: 505–523. doi: 10.1016/j.ajog.2013.05.006 23659989

[7] Ranjit E, Raghubanshi BR, Maskey S, Parajuli P. Prevalence of Bacterial Vaginosis and Its Association with Risk Factors among Nonpregnant Women: A Hospital Based Study. International Journal of Microbiology. 2018;2018: 1–9. doi: 10.1155/2018/8349601 29692813 PMC5859802

[8] Klatt TE, Barnabei VM. Factors Associated with Recurrent Bacterial Vaginosis. The Journal of Reproductive Medicine. 2010;55. 20337209

[9] Allsworth JE, Peipert JF. Severity of bacterial vaginosis and the risk of sexually transmitted infection. American Journal of Obstetrics and Gynecology. 2011;205: 113.e1–113.e6. doi: 10.1016/j.ajog.2011.02.060 21514555 PMC3156883

[10] Van Oostrum N, De Sutter P, Meys J, Verstraelen H. Risks associated with bacterial vaginosis in infertility patients: a systematic review and meta-analysis. Human Reproduction. 2013;28: 1809–1815. doi: 10.1093/humrep/det096 23543384

[11] Tang Y, Chen Y, Feng H, Zhu C, Tong M, Chen Q. Is body mass index associated with irregular menstruation: a questionnaire study? BMC Women’s Health. 2020;20: 226. doi: 10.1186/s12905-020-01085-4 33032583 PMC7545932

[12] Zhou X, Yang X. Association between obesity and oligomenorrhea or irregular menstruation in Chinese women of childbearing age: a cross-sectional study. Gynecological Endocrinology. 2020;36: 1101–1105. doi: 10.1080/09513590.2020.1803823 32783549

[13] Glueck CJ, Goldenberg N. Characteristics of obesity in polycystic ovary syndrome: Etiology, treatment, and genetics. Metabolism. 2019;92: 108–120. doi: 10.1016/j.metabol.2018.11.002 30445140

[14] Broughton DE, Moley KH. Obesity and female infertility: potential mediators of obesity’s impact. Fertility and Sterility. 2017;107: 840–847. doi: 10.1016/j.fertnstert.2017.01.017 28292619

[15] Poston L, Caleyachetty R, Cnattingius S, Corvalán C, Uauy R, Herring S, et al. Preconceptional and maternal obesity: epidemiology and health consequences. The Lancet Diabetes & Endocrinology. 2016;4: 1025–1036. doi: 10.1016/S2213-8587(16)30217-0 27743975

[16] Song X, Wang C, Wang T, Zhang S, Qin J. Obesity and risk of gestational diabetes mellitus: A two-sample Mendelian randomization study. Diabetes Research and Clinical Practice. 2023;197: 110561. doi: 10.1016/j.diabres.2023.110561 36738839

[17] Lokken EM, Richardson BA, Kinuthia J, Mwinyikai K, Abdalla A, Jaoko W, et al. A Prospective Cohort Study of the Association Between Body Mass Index and Incident Bacterial Vaginosis. Sexual Trans Dis. 2019;46: 31–36. doi: 10.1097/OLQ.0000000000000905 30148757 PMC6289672

[18] Bulut Z, Gazel D, Koç E. Investigation of the Relationship Between Obesity and Bacterial Vaginosis Using Microbiological Methods and Anthropometric Measurements. TMCD. 2020 [cited 8 Dec 2023]. doi: 10.5222/TMCD.2020.218

[19] Daubert E, Weber KM, French AL, Seidman D, Michel K, Gustafson D, et al. Obesity is associated with lower bacterial vaginosis prevalence in menopausal but not pre-menopausal women in a retrospective analysis of the Women’s Interagency HIV Study. Spradley FT, editor. PLoS ONE. 2021;16: e0248136. doi: 10.1371/journal.pone.0248136 33684141 PMC7939367

[20] Si J, You HJ, Yu J, Sung J, Ko G. Prevotella as a Hub for Vaginal Microbiota under the Influence of Host Genetics and Their Association with Obesity. Cell Host & Microbe. 2017;21: 97–105. doi: 10.1016/j.chom.2016.11.010 28017660

[21] Raglan O, MacIntyre DA, Mitra A, Lee YS, Smith A, Assi N, et al. The association between obesity and weight loss after bariatric surgery on the vaginal microbiota. Microbiome. 2021;9: 124. doi: 10.1186/s40168-021-01011-2 34049596 PMC8164250

[22] Brookheart RT, Lewis WG, Peipert JF, Lewis AL, Allsworth JE. Association between obesity and bacterial vaginosis as assessed by Nugent score. American Journal of Obstetrics and Gynecology. 2019;220: 476.e1–476.e11. doi: 10.1016/j.ajog.2019.01.229 30707966 PMC7232937

[23] Ouchi N, Parker JL, Lugus JJ, Walsh K. Adipokines in inflammation and metabolic disease. Nat Rev Immunol. 2011;11: 85–97. doi: 10.1038/nri2921 21252989 PMC3518031

[24] Hotamisligil GS. Inflammation, metaflammation and immunometabolic disorders. Nature. 2017;542: 177–185. doi: 10.1038/nature21363 28179656

[25] Sadeghi HM, Adeli I, Calina D, Docea AO, Mousavi T, Daniali M, et al. Polycystic Ovary Syndrome: A Comprehensive Review of Pathogenesis, Management, and Drug Repurposing. IJMS. 2022;23: 583. doi: 10.3390/ijms23020583 35054768 PMC8775814

[26] Srinivasan S, Liu C, Mitchell CM, Fiedler TL, Thomas KK, Agnew KJ, et al. Temporal Variability of Human Vaginal Bacteria and Relationship with Bacterial Vaginosis. Ratner AJ, editor. PLoS ONE. 2010;5: e10197. doi: 10.1371/journal.pone.0010197 20419168 PMC2855365

[27] Hickey R, Abdo Z, Zhou X, Nemeth K, Hansmann M, Osborn T, et al. Effects of tampons and menses on the composition and diversity of vaginal microbial communities over time. BJOG. 2013;120: 695–706. doi: 10.1111/1471-0528.12151 23398859

[28] Freeman EW, Sammel MD, Lin H, Gracia CR. Obesity and reproductive hormone levels in the transition to menopause. Menopause. 2010;17: 718–726. doi: 10.1097/gme.0b013e3181cec85d 20216473 PMC2888623

[29] Thoma ME, Klebanoff MA, Rovner AJ, Nansel TR, Neggers Y, Andrews WW, et al. Bacterial Vaginosis Is Associated with Variation in Dietary Indices,. The Journal of Nutrition. 2011;141: 1698–1704. doi: 10.3945/jn.111.140541 21734062 PMC3159055

